# The other side of the crisis: organizational flexibility in balancing Covid-19 and non-Covid-19 health-care services

**DOI:** 10.1186/s12913-022-08486-1

**Published:** 2022-08-29

**Authors:** Roberta Troisi, Stefania De Simone, Maria Vargas, Massimo Franco

**Affiliations:** 1 Department of Political and Communication Science, University of Salerno, Via Giovanni Paolo II, Fisciano (Salerno), Italy; 2 Department of Political Sciences, University of Naples Federico II, Largo S. Marcellino, Naples, Italy; 3 Department of Neurosurgical, Reproductive and Odontostomatological Sciences, University of Naples Federico II, Via Pansini, Naples, Italy; 4 Department of Political Sciences, University of Naples Federico II, Largo S. Marcellino, Naples, Italy

**Keywords:** Organizational flexibility, Capacity management, Demand management, Health crisis, Covid-19/no-Covid-19 patients

## Abstract

**Background:**

Many healthcare systems have been unable to deal with Covid-19 without influencing non-Covid-19 patients with pre-existing conditions, risking a paralysis in the medium term. This study explores the effects of organizational flexibility on hospital efficiency in terms of the capacity to deliver healthcare services for both Covid-19 and non-Covid-19 patients.

**Method:**

Focusing on Italian health system, a two-step strategy is adopted. First, Data Envelope Analysis is used to assess the capacity of hospitals to address the needs of Covid-19 and non-Covid-19 patients relying on internal resource flexibility. Second, two panel regressions are performed to assess external organizational flexibility, with the involvement in demand management of external operators in the health-care service, examining the impact on efficiency in hospital capacity management.

**Results:**

The overall response of the hospitals in the study was not fully effective in balancing the needs of the two categories of patients (the efficiency score is 0.87 and 0.58, respectively, for Covid-19 and non-Covid-19 patients), though responses improved over time. Furthermore, among the measures providing complementary services in the community, home hospitalization and territorial medicine were found to be positively associated with hospital efficiency (0.1290, *p* < 0.05 and 0.2985, *p* < 0.01, respectively, for non-Covid-19 and Covid-19 patients; 0.0026, *p* < 0.05 and 0.0069, *p* < 0.01, respectively, for non-Covid-19 and Covid-19). In contrast, hospital networks are negatively related to efficiency in Covid-19 patients (-0.1037, *p* < 0.05), while the relationship is not significant in non-Covid-19 patients.

**Conclusions:**

Managing the needs of Covid-19 patients while also caring for other patients requires a response from the entire healthcare system. Our findings could have two important implications for effectively managing health-care demand during and after the Covid-19 pandemic. First, as a result of a naturally progressive learning process, the resource balance between Covid-19 and non-Covid-19 patients improves over time. Second, it appears that demand management to control the flow of patients necessitates targeted interventions that combine agile structures with decentralization. Finally, untested integration models risk slowing down the response, giving rise to significant costs without producing effective results.

## Background

Health-care systems are facing major challenges because of Covid-19 pandemic. Over the two years of the pandemic, the rapid spread of the virus has undermined the ability of most health systems to provide an adequate response, with implications for Covid-19 and non-Covid 19 patients [1]. Many health systems appear to have been unable to cope with the pandemic while safeguarding non-Covid-19 patients with pre-existing conditions. In the hospitals, the urgent need to contain the pandemic led to a reallocation of resources, with the transfer of medical and nursing staff from other areas of the health-care service, increasing the provision of beds for Covid-19 patients while at the same time reducing those for other patients, and saturating intensive care units with Covid-19 patients [2]. This imbalance between Covid-19 patients and those with a range of other needs inevitably has serious implications for the well-being of non-COVID 19 patients [3, 4], and particularly in the case of chronic and oncological patients, increasing the risk of death [5, 6]. Health systems that prioritize the pandemic while failing to respond adequately to the other conditions not related to COVID 19 run the risk of paralysis in a medium-term perspective [7].

Given the uncertainty about the duration of a pandemic, the critical challenge is to balance the requirements of COVID-19 and non-COVID-19 patients, ensuring adequate access to resources and facilities for both groups. Health systems, particularly hospitals, have to adapt to these changing needs, reconfiguring the provision of services.

The question to be addressed is whether and how healthcare services have made changes in terms of structural design and the use of their resource portfolio to manage the two different sets of health-care needs. This paper aims to examine the influence of organizational flexibility in managing the two distinct sets of needs.

Flexibility is necessary for reconfiguration since it can allow an enhanced capacity to manage both sets of needs, avoiding excessive imbalances, and, where possible, respond to the two sets of needs within a reasonable timeframe. The theoretical framework is based on insights from organizational studies. In particular, structural contingency theory indicates that organizations that promptly address environmental uncertainty are likely to be more effective [8, 9]. This means providing a response based on an ability to adapt to external changes, the extent of which is recognized in the literature as the degree of flexibility that characterizes the organization of the system, as well as its parts [10]. In our case, this means dealing with health systems as a whole and, in particular, hospitals as key service providers. Despite the differences between health systems, complementary actors or organizations typically play a role as healthcare providers that is secondary to that of hospitals [11].

Among the subtypes described in the literature, two kinds of flexibility are in line with the aims of this study. The first concerns *resource flexibility*, dealing with the ability to dynamically reallocate resources while strengthening them [12]. This study highlights the internal dimension of hospitals to explain their capacity to effectively manage two competing sets of needs. In contrast with resource flexibility, *structural flexibility* concerns the ability to integrate, build and reconfigure the design of the system [13]. We use this sub-type of flexibility as external to hospitals, considering the degree of involvement of other actors within the health system with a view to smooth the flow of the two competing sets of needs.

Hospital capacity management is considered efficient when it can deliver health care services for both sets of needs. At the same time, it is conditional on external demand management for smoothing the flow of the two sets of needs, thus helping to find a balance between the two categories of hospitalized patients. This measure of “organizational efficiency” is in line with that of Pfeffer and Salanick [14] which considers it to be an external standard of how well an organization is meeting demands of various groups.

A two-step strategy is adopted. First, an assessment is made of the capacity of hospitals to efficiently manage the two sets of needs by relying on internal resource flexibility, carrying out a Data Envelope Analysis. Second, two panel regressions are carried out to verify whether external organizational flexibility, expressed in terms of the involvement of other health-care service providers in demand management, impacts on hospital capacity management.

The case examined is the Italian National Health Service, which offers public access to health-care on a regional basis, with the regional authorities responsible for the organization and delivery of health-care services based on national funding. Italian National Health Service has been severely depleted over the past 10 years by cuts in public spending, with organizational consequences in terms of reduced capacity to respond to current needs [15], which is significantly below the European average, and characterized by stark geographical differences between the northern, the central and especially the southern regions [16]. It is, therefore, interesting to examine the response capacity of a system that was already significantly depleted, while considering that, initially, there has been a greater spread of the virus in the best-performing northern regions.

In the following sections, insights from the literature concerning organizational flexibility are highlighted, then the models are described, and the results are outlined and discussed. In the conclusions, the practical implications of the study are examined.

## Theoretical considerations: flexible responses in an ever-changing environment

Health-care systems have been described as complex adaptive systems due to the speed of change of patients’ needs [17], both at local and global level, considering that the demand is increasing in qualitative and quantitative terms, giving rise to the need for greater competitiveness [18]. Researchers identify one way to address this unpredictability in terms of flexible organizational solutions to adjust to the ever-changing environment. Studies of organizational flexibility indicated various sets of flexibility determinants with two that are particularly relevant in effectively responding to environmental challenges: combination of resources [19] and the structural configuration [20].

### Resource flexibility

Resource flexibility is often linked to capacity management that is primarily concerned with ensuring that the organization can respond appropriately to the level of expected demand [21]. Capacity management involves decision-making relating to the allocation of key resources that in the health-care sector consists of facilities, medical equipment, technologies, and health processes, considering the state of continuous fluctuation of the environment [22]. Resource flexibility consists of the ability to use specific resource compared to a wider range of alternatives. In addition, it can concern the ability to modify the total amount of resources [23]. Resource flexibility is an advantage for two main reasons: first, it allows a fast response to rapid changes in uncertain environments; second, switching the application of resources from one use to another can partially overcome the lack of resources typically affecting an organization, especially in health-care systems where the optimal use of limited resources is challenging due to the spending restrictions placed on the public sector [24].

When referring to organizational activities, the optimal use of resources involves a range of activities outside the organization and others intended to ensure regular behaviour, supporting the combination of both proactive and reactive responses [25]. Similarly, when referring to strategy, it has been argued that an optimal balance between strategic options involve the use of flexible resources by different measures together with those focused on particular actions [26]. Empirical studies have shown that resource flexibility contributes positively and significantly to improve the performance of health-care services in terms of capacity. In particular, for the highest-ranking performers with regard to capacity management, a positive correlation has been found between highly turbulent contexts and high levels of resource flexibility. In a complementary way, resource flexibility has been measured in terms of cost-effectiveness: flexibility makes sense when it entails reasonable changes to the existing combinations of resources at an affordable cost [27]. The focus on the imbalance between flexible and fixed resources has been explored mainly in theoretical terms for its implications for the performance of health-care services. For example, it has been argued that in a situation where the level of the demand exceeds the capacity of hospitals to deliver health-care services, organizational efficiency will depend on the capacity to protect internal routes with a buffer of focused resources and to proactively deploy flexible resources using various routes according to the type of excess demand [28].

We propose an empirical assessment of the efficiency of capacity management in a particular case of excess demand due to a health emergency.

Based on the arguments outlined above, hospitals need to attempt to achieve efficient capacity management through an optimal combination of resources to deal with the emergency and at the same time ensuring an adequate level of regular services.

### Structural flexibility

Structural flexibility often goes together with demand management, since these efforts typically aim to address an increase in demand by redirecting resources through structural reconfigurations [29, 30]. In turbulent contexts, the organizational flexibility necessary to survive was not considered when the organization was designed. Many types of organizational design have been proposed to achieve flexibility. Lawrence and Lorsch [31] were pioneers in describing the need to increase the degree of autonomy of peripheral units from core units according to the level of environmental uncertainty. In this connection, several models have been identified in the literature, including decentralized decision-making, low levels of formalization, horizontal integration, and collaborative partnerships [13].

In the healthcare sector, organizational flexibility concerns the relations between hospitals and various agents within health-care systems, highlighting the need for integration and/or differentiation between internal (core services) and external activities of a complementary type [9]. The focus is on demand management to control the variability of health-care needs and to match them efficiently with the available capacity [27]. Specifically, integration is described as auxiliary organizational flexibility. Reducing demand leads to a particular unit (typically hospitals) focusing on a set of essential facilities by transferring auxiliary facilities to other providers. The degree of integration among providers may increase due to the amount and kind of facilities supplied. Thus, this integration model includes informal and formal collaboration, outsourcing and networking [32].

As for differentiation, various studies highlight the autonomy required by the need to respond quickly to uncertainty in the level of demand that may be specific to the circumstances [33]. This decentralized response to the uncertainty arising from a particular set of needs prioritizes focused activity with weak links with the centre, albeit within a shared objective of controlling common demand [34]. Finally, when measuring the impact of organizational flexibility on performance, the studies point to its cost-efficiency [35].

This is evident because although reconfiguration models vary in degree, they often require an economic effort at the development stage. Flexibility is considered key in determining efficient demand management, and, it has been seen as a means to manage peak demand and to address a certain amount of volume flexibility [28].

Demand management within the health system should positively impact hospitals’ capacity to treat Covid-19 and non-Covid-19 patients. As a result, it is a challenge to identify the right type and level of system flexibility that can provide adequate support for hospitals dealing with a critical increase in demand.

**Fig. 1:**
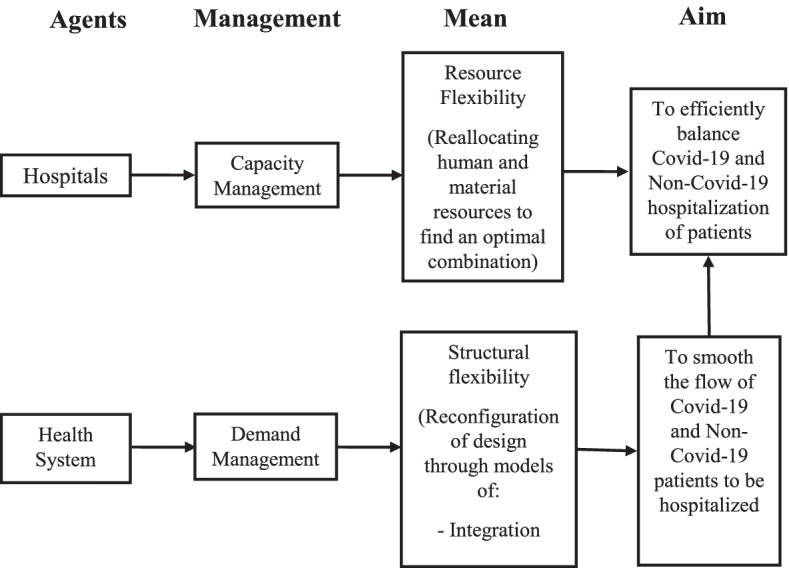
Study design

Figure 1 shows the study design, highlighting the role of the flexibility both for capacity and demand management.

## Methods

This section describes the data and details of the empirical methods. We used a two-step analysis. The first step aimed to assess hospitals’ capacity to handle both types of health-care demands (Covid-19 and non-Covid-19). We used Data Envelope Analysis to measure technical efficiency based on internal resource flexibility. In the second step, two panel regressions were used to investigate whether external organizational flexibility within regional health systems had a significant impact on the technical efficiency of hospitals.

**Fig. 2:**
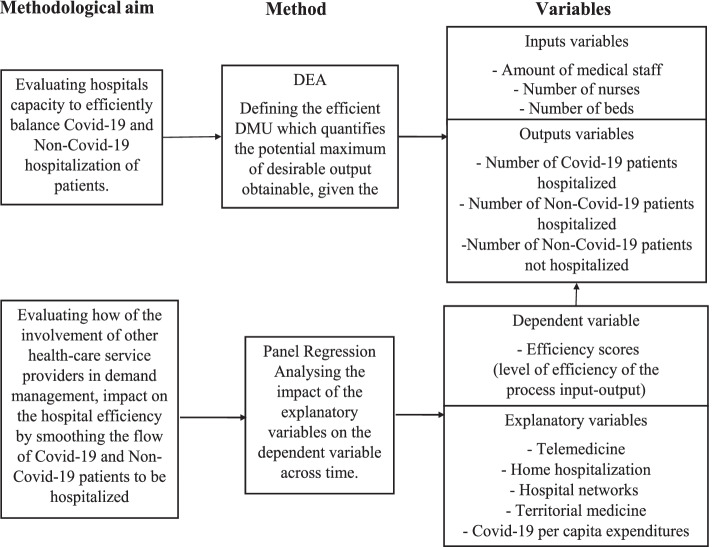
Methodological work-flow

Figure 2 shows the methodological work-flow.

### Data sources and sample

In the first step, the data concerning input variables to express internal flexibility were retrieved as follows. The website of the Italian Ministry of Health [36] provides detailed and updated information about hospital resources, medical and non-medical staff, and the number of beds at a regional level. In addition, the Italian Court of Auditors [37] provide exhaustive information about the number of medical and non-medical staff specifically employed in managing Covid-19 patients on a regional basis. The number of hospitalized Covid-19 and non-Covid-19 patients was retrieved from the Ministry of Health [36] and AGENAS websites [38].

In the second step, data on the delivery of health-care facilities through telemedicine, home hospitalization, and hospital networks were retrieved from Ministry of Health website. In addition, the data on territorial medicine (see Step 2: model and variables) were retrieved from the regional ordinances available on the official regional websites. Finally, the annual report of the Court of Auditors provides information on the per capita health-care expenditure of the regional authorities, specifying the amount relating to Covid-19 health-care expenditure.

### Step 1: model and variables

In the first step Data Envelope Analysis (DEA) was performed. DEA is a non-parametrical tool for assessing efficiency. Specifically, the model makes it possible to define the efficient Decision-Making Units (DMUs) among the DMUs analysed, in our case hospitals, as key units within the regional health-care system. Using linear optimization, the efficient DMU identifies the maximum potential output obtainable, given the number of inputs in which the DMU operates efficiently (output-oriented). Substantially it compares the average efficiency of each group of the regional hospitals rather than trying to estimate efficiency thresholds. The model is widely used in studies dealing with health-care efficiency [39–41].

We utilized the linear programming system [42]. This particular model projects the observations in an assigned direction, consenting to take counts of undesirable outputs inevitably linked to the production. Frontier units are specifically defined as DMUs that maximize the desired output while concurrently minimizing the undesirable one given a fixed amount of input. The efficiency is valuated solving the following linear model:

$$\underset{\beta ,\lambda }{\mathrm{min}}\beta$$

$$s.t. X\lambda \ge {x}_{i}$$

$${Y}^{d}\lambda \ge {y}_{o}^{d}+\beta {y}_{o}^{d}$$

$${Y}^{u}\lambda \le {y}_{o}^{u}-\beta {y}_{o}^{u}$$

$$\mathrm{max}\{{y}_{i}^{u}\}\ge {y}_{o}^{u}-\beta {y}_{o}^{u}$$

$$\lambda \ge 0$$

where $X$, ${Y}^{d}$ and ${Y}^{u}$ are, respectively, the inputs and the desirable and undesirable outputs matrix, $\lambda$ is the optimal weight vector and $\beta$ is the optimal solution of the problem. A DMU is considered on the frontier, then directional efficient, when $\beta =0$ and ${\lambda }_{0}=1$, ${\lambda }_{j}=0$ ($o\ne j)$. The analysis was performed using the Matlab Toolbox developed by [43].

A DMU is typically considered efficient if it receives a score of 1.00, which denotes 100% efficiency, whereas a score of less than 1 denotes inefficiency. This suggests that the linear system determines relative, rather not absolute, efficiency rankings. Despite receiving a score of 100% efficiency, DMU on the efficient frontier probably have possibility to increase their output.

The widespread use of this method is due to its advantages. First, it is not necessary to define the formal relationship between the input and output variables considered. This results in a simpler model specification, especially when the relationship between inputs and outputs is complex or unknown. Second, it can be used with a small number of DMUs and considering multiple inputs and outputs. In the present study, we performed several DEAs distinguishing two different types of demand for services that regional health care systems have to deal with: Covid-19 and non Covid-19. In addition, in Covid-19 crisis [44] demand is not constant over time. In the next step, we distinguished between three phases of demand: from March 2020 to June 2020, coinciding with the first surge of the pandemic; from July 2020 to September 2020, when demand of Covid-19 patients declined; from October 2020 to December 2020, coinciding with the second surge of the pandemic. The timeframe for the analysis was up till December 2020 as not all the data required for the analysis of 2021 had been made available at the time of writing. The decision to use surges as a measure of each phase’s duration to compare with variations in patient demand respect the pandemic trend. As a result, variations in hospitalized patients are estimated based on the trend of COVID-19’s spread. This period is used in many studies that examine the effects of covid-19 during the two years of the virus’s spread, including its mortality [45], the incidence on patients suffering from other diseases [46], and the quality of polluted cities [47]

In the analysis, the resources employed for meeting each type of demand were considered as inputs. Since resource flexibility reflects the combination of available resources, the analysis makes it possible to establish whether hospitals efficiently used this kind of flexibility to meet the needs of the two categories of patients, and thus striking a balance among the patients’ needs. The results were aggregated on a regional basis corresponding to each regional health systems. The inputs were defined and operationalized as follows.

1. The amount of medical staff. The variable is used in most health-care efficiency studies [39, 41, 48]. In particular, in the case of the demand for treatment for Covid-19 patients, the total number of medical staff in each phase was given by the medical staff taken from non-Covid-19 services, plus the medical staff specifically employed during the pandemic for Covid-19.
2. The number of nurses. This variable is considered in studies on hospital efficiency [48, 49]. As for the medical staff, we considered the number of nurses assigned to Covid-19 and non-Covid-19 patients.
3. The number of beds. This variable is typically used as a proxy for hospital size and capital investment [39].

The output (variable) is defined by the number of hospitalized patients [50]. In particular, for Covid-19, for each phase, we considered the number of patients hospitalized for Covid-19. At the time of writing, no information was available about how many of these cases are Covid-19 patients hospitalized for other reasons.

For non-Covid-19 patients, we considered two outputs: one desirable and one undesirable [51]: 1) the number of hospitalized non-Covid-19 patients, considered as the desired output; 2) the number of non-hospitalized non Covid-19 patients. This variable considers the non-Covid-19 patients ‘needs that hospitals were unable to meet. The variable is operationalized as the difference between the average number of hospitalized patients in the three years prior to 2020, for the period relating to each phase, and the number of hospitalized patients during the pandemic, in line with the procedure adopted by the Court of Auditors to evaluate patient backlog. The linear optimization for the evaluation of efficiency considers this variable as an output to be minimized. Table 1 outlines the variable used in the DEA models.

**Table 1:** Variables used in the DEA models

Variable	Type	Model
The number of medical staff	Input variable	Non-Covid-19 and Covid-19
The number of nurses	Input variable	Non-Covid-19 and Covid-19
The number of beds	Input variable	Non-Covid-19 and Covid-19
Number of patients hospitalized for Covid-19	Desirable output variable	Covid-19
The number of hospitalized non-Covid-19 patients	Desirable output variable	Non-Covid-19
The number of Non-Covid-19 non-hospitalized patients	Undesirable output variable	Non-Covid-19

### Step 2: model and variables

The second step of the analysis was the implementation of two random-effect panel models. This analysis investigates the relationship over time between a set of explanatory variables for organizational flexibility and the efficiency scores obtained in the first step, as the dependent variable. Analytically the model can be expressed as follows:

1 $$y_{it}=\alpha+\beta_{it}\;x_{it}+u$$

where *y*_*it*_ is the dependent variable of interest at time *t*, in the present study the efficiency scores of the hospitals within the regional health systems. On the other hand, *x*_*it*_ is a set of time-varying explanatory variables, whereas α, and *β*_*it*_ are, respectively, the intercept and the parameters to be estimated, while *u* is the idiosyncratic error term.

Four measures taken on a regional basis to respond to the pandemic are considered proxies for organizational flexibility and, as such, are the explanatory variables of interest.

They were all implemented or intensified during the period considered, whereas previous measures of similar type are excluded since they cannot be reasonably expected to be relevant to the management of the two sets of needs.

Telemedicine consists of the delivery of health-care and the exchange of health-care information across distances [52]. The advantages consist of providing care not previously deliverable, particularly useful in times of emergency for the accessibility that it can ensure. During the Covid-19 pandemic, it facilitated compliance with social distancing [53]. In our study, it is taken for proxy of external organizational flexibility for two reasons. First, it helps to define organizational flexibility in response to the variability of the needs of patients It constitutes a flexible unit of health-care services architecture combining real and virtual spaces [54]. Second, it is considered external to hospitals since in Italy, telemedicine was mainly managed by health-care service units rather than hospitals, to reduce the flow of hospitalized patients [36]. Finally, many studies provide evidence of the positive impact on health-care efficiency, mainly in terms of increased access rates, number of patients examined per hour, and patient experience of care [55–58]. This measure is expressed in terms of the number of telemedicine consultations on a regional basis in the period of analysis.

Home hospitalization refers to care provided at home by social and health-care professionals. It has different features that vary from country to country. In some cases, this type of care is provided by the public sector aimed at decentralizing and strengthening health-care services other than hospitals. In other cases, private-sector services are provided through outsourcing or collaborative partnerships. However, it ensures a measure of external organizational flexibility as it reconfigures the health architecture, aiming to mitigate the hospital-centric character of the health system, employing a range of staff and facilities to reduce the number of in-patients [59]. During the period under examination, Italy set up a system of Special Continuity Care Units (USCA) established on a regional basis, with newly hired staff or health-care workers seconded from hospitals to provide health facilities for non-critical patients, not limited to those with Covid-19. Home hospitalization is a cost-effective alternative to conventional hospitalization for selected patients. In addition, some studies have shown a positive impact on quality of care, and patient satisfaction [60]. It is measured here as the percentage of special units established in relation to those expected for the population.

Hospital networks consist of multihospital systems based on horizontal integration to improve service delivery and reduce variability by complementing services through the network [61]. The aim is to increase capacity to respond to uncertainty in demand for health-care services [62]. During the Covid-19 pandemic this resulted in a better allocation of patients, making possible an improvement in configuration in terms of the workload among hospitals in the network [63]. Hospital networks have been considered as a proxy for external organizational flexibility in many studies to expand capacity and, at the same time, rapidly adapt to changing patient needs. The literature highlights how adopting hospital networks can improve hospitals’ overall performance due to their capacity to foster innovation [64]. This variable is dichotomous, assuming a value of one if the network is used, and zero otherwise.

Territorial medicine is regulated by Italian law as a model of horizontal integration within health-care systems. During the Covid-19 pandemic, it mainly consisted of social and healthcare units (such as rehabilitation facilities or clinics for long-stay patients) converted into temporary Covid-19 units [2]. The detailed framework is defined on a regional basis, whereas the management is the responsibility of the local health authorities (ASL). These facilities can be used to admit non-critical Covid-19 patients.

To the best of our knowledge, no previous studies have been published on this way of providing care, it can easily be considered a form of external flexibility that brings together facilities and medical teams working outside hospitals. It is measured in this study as the number of health-care units converted into temporary Covid-19 units.

Finally, per capita public expenditure for Covid-19 was measured as control variable. Previous results do not provide conclusive evidence since some studies show no correlation between public expenditure and the ability to contain Covid-19 [44, 65], whereas other studies report lower Covid-19 fatalities in countries with significant resources dedicated to health-care [66].

Table 2 outlines the variables used in the panel regression model.

**Table 2:** Variables used in the Panel regression model

Variable	Type	Description	Measurement
Hospitals Efficiency scores	Dependent variable	The efficiency scores obtained in the first step of the analysis through a DEA model	Adimensional. Assumes value 1 if 100% efficient, less than 1 if inefficient
Telemedicine	Exploratory variable	The delivery of health-care and the exchange of health-care information across distances	The number of telemedicine consultations on a regional basis in the period of analysis
Home hospitalization	Exploratory variable	Health care services provided at home by Special Continuity Care Units (USCA)	The percentage of special units established in relation to those expected for the population
Hospital networks	Exploratory variable	Multihospital network systems	Assumes value 1 if the network is used, 0 otherwise
Territorial medicine	Exploratory variable	Social and healthcare units (such as rehabilitation facilities, or clinics for long-stay patients) converted into temporary Covid-19 units	The number of health-care units converted into temporary Covid-19 units
Covid-19 per capita expenditure	Control variable	Per capita public expenditure for Covid-19	Euros/inhabitants

## Results

This section presents the results of the model outlined in the previous section. Table 3 shows the complete set of results for the DEA models, while Table 4 summarizes the results of the panel regression model. Table 3 presents the analysis of hospital capacity management efficiency (hereinafter, hospital efficiency) on a regional basis, distinguishing the results for the three phases of the pandemic (columns 1–3 for non-Covid-19, columns 4–6 for Covid-19). Results show an average hospital efficiency of 0.87 and 0.58, respectively, for Covid-19 and non-Covid-19 patients. This suggests that, on average, for Non-Covid-19 and Covid-19 patients, respectively, a score of 28% and 42% higher is needed to achieve the frontier of efficiency (100%). For instance, Abruzzo reached a non-Covid-19 Hospital efficiency score of 0.80 (80%) during the first phase, indicating that an increase of the 20% of patients admitted, given the same number of resources, is necessary to reach the frontier of efficiency. The table shows that the efficiency score frequently falls below 0.75, indicating a relatively high level of inefficiency. From a practical point of view this means that the health systems should increase all of its outputs by 25% to become -relatively- efficient [67].

**Table 3:** DEA results

Regions	Non Covid-19 Hospital efficiency			Covid-19 Hospital efficiency		
	Phase 1	Phase 2	Phase 3	Phase 1	Phase 2	Phase 3
Abruzzo	0.84	0.83	0.93	0.30	0.37	0.69
Basilicata	0.75	0.68	0.77	0.57	0.61	0.58
Calabria	0.79	0.83	0.32	0.14	0.21	0.29
Campania	0.90	1.00	1.00	0.07	0.27	0.48
Emilia Romagna	0.91	0.95	1.00	0.45	0.36	0.30
Friuli Venezia Giulia	0.93	0.86	1.00	0.28	0.45	0.89
Lazio	1.00	1.00	1.00	0.11	0.29	0.39
Liguria	0.93	1.00	1.00	0.75	1.00	1.00
Lombardia	1.00	1.00	0.92	1.00	1.00	1.00
Marche	0.80	0.89	1.00	0.39	0.37	0.52
Molise	0.69	0.98	1.00	1.00	1.00	1.00
PA Bolzano	0.97	0.91	1.00	0.58	1.00	1.00
PA Trento	0.90	0.82	0.90	1.00	1.00	1.00
Piemonte	1.00	1.00	1.00	1.00	1.00	1.00
Puglia	0.81	0.90	0.41	0.11	0.16	0.19
Sardegna	0.81	0.76	0.89	0.13	0.30	0.28
Sicilia	0.86	0.80	0.90	0.08	0.39	0.68
Toscana	0.87	0.76	0.95	0.20	0.24	0.45
Umbria	0.83	0.72	0.81	0.22	0.47	0.75
Valle d'Aosta	0.76	0.68	0.94	1.00	1.00	1.00
Veneto	1.00	1.00	0.94	0.46	0.77	0.74
Italy	0.87	0.87	0.89	0.47	0.58	0.68
North	0.93	0.91	0.97	0.72	0.84	0.88
Centre	0.83	0.88	0.95	0.41	0.50	0.67
South	0.82	0.83	0.72	0.18	0.32	0.42

**Table 4:** Panel regression results

Exploratory variable	Non Covid-19 Hospital efficiency	Covid-19 Hospital Efficiency
Intercept	0.7841*** (0.0382)	0.3302*** (0.0721)
Telemedicine	0.0018* (0.0007)	-0.0002 (0.0014)
Home Hospitalization	0.1290* (0.0610)	0.2985** (0.1054)
Hospital Networks	-0.0242 (0.0275)	-0.1037* (0.0500)
Territorial Medicine	0.0026* (0.0013)	0.0069** (0.0022)
Covid-19 per capita expenditure	-0.0005 (0.0003)	0.0006 (0.0006)
R-squared	0.40	0.41
Wald-test	*P* < 2.7e-09	*P* < 5.8e-09

Breusch-Pagan test for balanced panels found a significant panel effect (*p* < 0.001)

Multicollinearity was tested using the Variance Inflation Factor (VIF). There is no evidence of multicollinearity, based on the recommended threshold of 5 for the VIF

Significance code: *** *p* < 0.001; ** *p* < 0.01; * *p* < 0.05

**Fig. 3:**
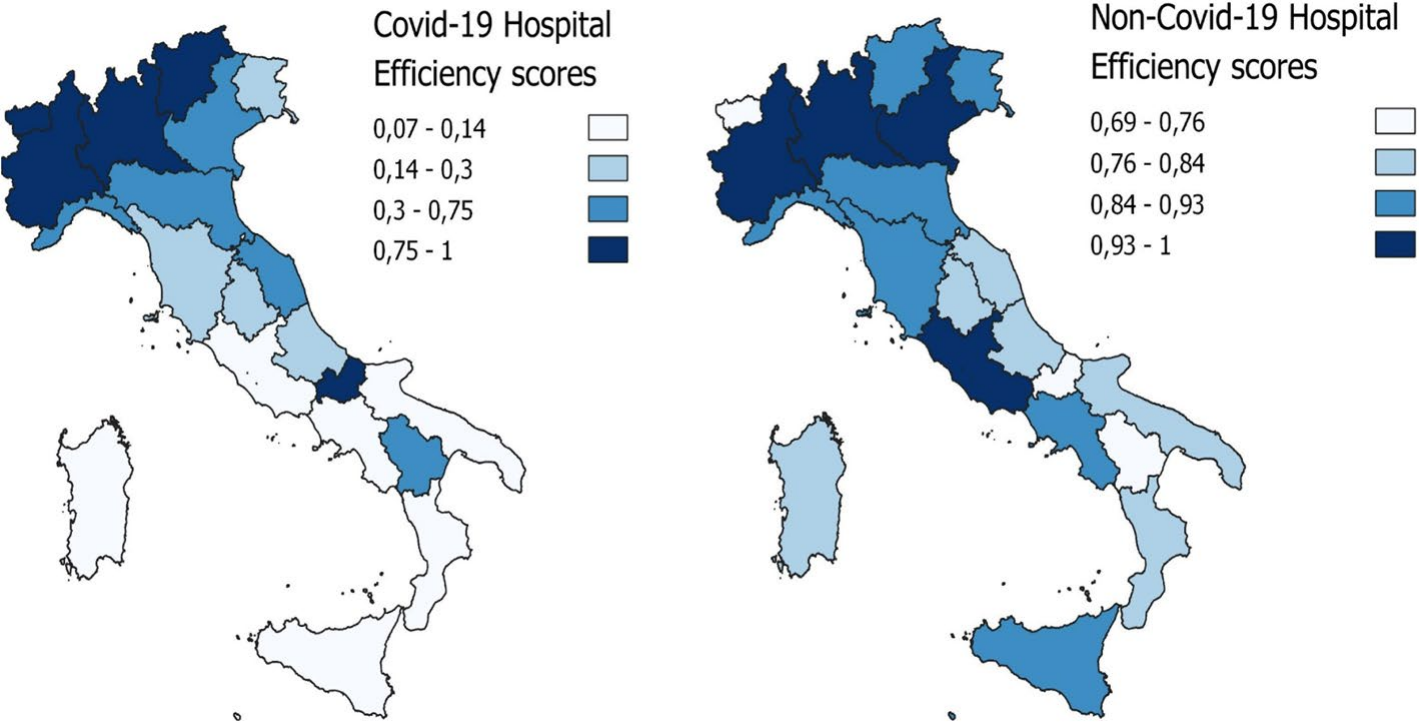
First phase DEA results

**Fig. 4:**
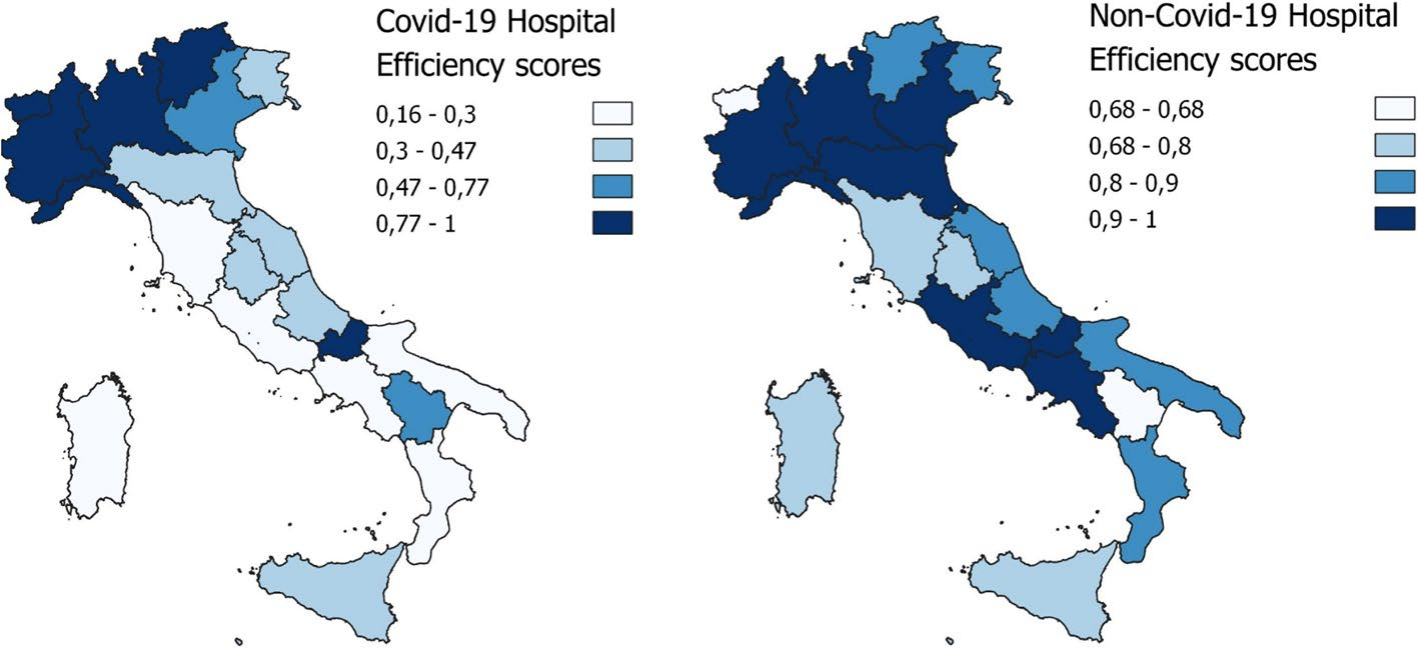
Second phase DEA result

**Fig. 5:**
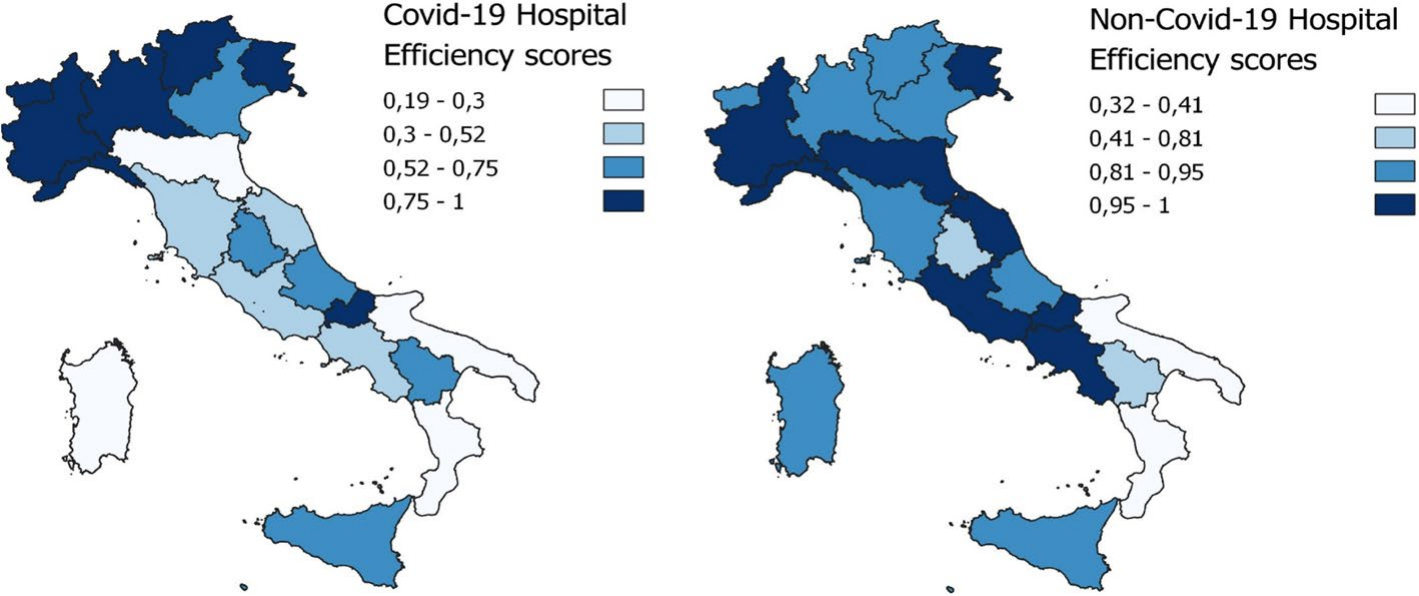
Third phase DEA results

By clustering based of geographical areas, the results highlight how the efficient Decision-Making Units (DMUs) are more highly concentrated in the northern regions, comparing Covid-19 and non-Covid-19 hospital efficiency for the entire period under examination. In addition, the efficiency results for the northern and central regions tend to improve for Covid-19 and non-Covid-19. However, the south shows the least favourable efficiency results in terms of dealing with the demands of the two categories of patients, and in particular a decline in the efficiency score for non-Covid-19 patients in the third phase, contrasting with a limited improvement in hospital efficiency for Covid-19 patients in the same phase. Table 3 displays the results, and Figs. 3, 4, and 5 map them geographically.

The results in Table 4 show that telemedicine is positively related (0.0018, *p* < 0.05) to the efficiency scores in the case of non-Covid-19 demand. Thus, introducing a greater number of telemedicine facilities seems to increase the efficiency of hospitals in responding to the needs of non-Covid-19 patients. As expected, home hospitalization is positively related to efficiency in both models (0.1290, *p* < 0.05 and 0.2985, *p* < 0.01, respectively, for non-Covid-19 and Covid-19 patients). This means that home hospitalization effectively supports hospitals dealing with the pandemic. In contrast, hospital networks are negatively related to efficiency in the case of Covid-19 patients (-0.1037, *p* < 0.05), while the relationship is not significant in the case of non-Covid-19 patients. As a result, the setting up of hospital networks was not found to be a particularly effective measure for dealing with Covid-19. Territorial medicine, as expected, has a positive effect on hospital performance (0.0026, *p* < 0.05 and 0.0069, *p* < 0.01, respectively, for non-Covid-19 and Covid-19. Finally, for both models, the control for Covid-19 per capita expenditure is not significant.

## Discussion

Our results show how the overall hospital response in the period examined was not fully efficient, as the analysis shows an average efficiency score less than one for both categories of patients. The healthcare system distributes resources for both categories of patients inefficiently. It is likely that the spending restrictions imposed on the public health sector and the resulting hospital’s reduced organizational capacity had an impact on the overall performance. The lower efficiency score in addressing the needs of Covid-19 patients (0.58) compared to non-Covid-19 patients (0.87) for the entire period under examination may seem surprising. However, these results deserve closer attention. First, Covid-19 is an unprecedented disease and the limited experience in treating Covid-19 patients affected hospital efficiency in treating these patients more than non-Covid-19 patients. Second, the efficiency analysis is not parametric, and as a result the “efficient” DMUs identified in the study should be interpreted as the benchmark or best-practice frontier, and the difference in efficiency is the distance from best practice. It should be noted that all the regions faced a level of demand for non-Covid-19 patients that they were unable to meet (in some cases greater than 30%) [38]. Efficient hospitals were the most effective at offsetting this undesirable effect due to the limited resources available. Some regions managed both categories of patients more efficiently by balancing the use of their resources. In particular, the northern regions had the highest efficiency scores simultaneously with regard to both categories of patients, implying a better distribution between reactive and proactive choices than southern regions. For example, Piedmont in the north is always on the efficiency frontier, whereas Apulia in the south is one of the least successful at meeting the needs of both categories of patients.

This finding reflects qualitative and quantitative differences in hospital services between northern and southern regions in the years preceding the pandemic. While austerity policies have affected the country, the northern regions have managed to ensure service accessibility and appropriateness. On the contrary, the southern regions have mainly targeted cost containment (for example, no new hires, reduction in the purchase of new machines) with obvious implications on the efficiency and quality of services [68]. The disparity in hospital services between the North and South has most likely been exacerbated by Covid-19, emphasizing the Southern Regional Health Service’s endemic limitations. In times of emergency, resource flexibility is not only a problem of redeployment but also highlights the urgent need for adequate resources to be reused.

In addition, the increased efficiency over time in managing Covid-19 for all regions, suggests a learning process in managing these patients that does not appear to be present in the management of non-Covid-19 patients.

For the second step, the results show measures, among those attributable to external flexibility, positively associated with the capacity of hospitals to meet the needs of Covid-19 and non-Covid-19 patients. This raises two questions: in terms of management, what helps to better control the demand flow that hospitals have to manage, and in terms of models, whether a particular design configuration can be identified for this purpose.

Telemedicine is positively associated with efficiency only regarding non-Covid-19 patients. This model is based on a high degree of autonomy that seems to work well when virtual support is provided for patients with well-known diseases. In the event of a new disease and limited knowledge, a more integrated model working in synergy with hospitals and field experience would probably work better [53].

In line with the existing literature, home hospitalization and territorial medicine are positively related to efficiency in both models [60]. Both are measures to strengthen peripheral units, that are endowed with a high degree of autonomy. Unlike telemedicine, the explanation of the ability to manage demand is related to two factors: first, these measures are focused on non-critical patients, and second, they require direct contact, resulting in a complementary health-care service that can be efficiently separated from hospital management [34].

In contrast with the existing literature [63, 64], hospitals networks are negatively related to hospital efficiency in the case of Covid-19, while the relationship is not significant in the case of non-Covid-19 patients. This is the only case of integration in a general framework of organizational flexibility in the information from the official sources, and in many cases the networks were in operation for the first time as they were set up specifically for Covid-19.

The joint effort of human resources, medical equipment, and technological support for optimal functioning probably requires a more extended timeframe than possible during a pandemic. Finally, the control for per capita expenditure for Covid-19 patients was not significant for either model. Thus, better results are not necessarily the outcome of increased expenditure. This argument points to the need for an overall interpretation of the results for two reasons. First, the regions that achieved better outcomes despite spending cuts prior the pandemic are better able to balance their resources in critical periods. Although balancing two sets of demands is unsatisfactory in terms of efficiency in the context of scarce resources, their balance improves over time with an improved understanding of what the pandemic requires. Second, some measures are more suitable than others to support hospital efficiency. This lends weight to the argument that adequate choices and appropriate objectives can make a difference in certain circumstances, even more than total expenditure.

Our study has limitations. The emphasis on resource flexibility in hospital capacity management and organizational flexibility in the management of demand external to hospitals, albeit within the health system, clearly captures only part of the overall picture, since it fails to examine internal organizational flexibility and external resource flexibility. However, this approach was adopted since it better represents health system responses to Covid-19. This highlights the urgency of identifying agile solutions on the part of hospitals, which often require reallocating resources such as staff or hospital beds instead of making more complex structural changes. On the other hand, organizational flexibility better captures system’s dynamics during a pandemic while examining how units other than hospitals have been involved in dealing with the emergency, thus reducing hospital congestion. Another limitation of the present study is that it does not directly consider the variability of the spread of the infection across the regions. Still, this can be considered a partial limitation since the number of hospitalizations is significantly correlated to the number of infections. In addition, this measure serves as a proxy for the seriousness of the cases since, in general, only the most serious Covid-19 cases require hospital treatment.

Further research based on a longer timeframe may be required to verify the long-term effects. In addition, the study shows how efficiency in managing the needs of the two categories of patients can be achieved if hospital resources are well distributed.

Future research should quantify the amount of resources assigned to each category of patients to achieve efficient responses within a reasonable timeframe.

## Conclusions

This study explores the effects of organizational flexibility on hospital efficiency intended as the capacity to deliver health-care services for both Covid-19 and non-Covid-19 patients. The focus is on Italian health systems and mainly on local services provided on a regional basis. The role of hospitals as critical units is underlined, followed by a discussion of internal flexibility concerning hospitals and external flexibility in relation to service providers other than hospitals within regional systems. Two main determinants of organizational flexibility were considered: resource flexibility, relating to the internal capacity of hospitals to combine their resources, and organizational flexibility, relating to external flexibility in smoothing the provision of services for the two categories of patients in terms of their hospitalization using different structural configurations. In relation to previous studies in this field, and particularly those that rely on flexibility as a measure of the organizational performance of health-care services, the contribution of the present study consists of bringing together external and internal flexibility measures at the same time, considered as essential to efficient hospital capacity management. The link between these two measures is the degree of flexibility that brings together different actors and different responses. Although the primary units of analysis are hospitals, a systemic approach was adopted that is essential in the context of a pandemic that is unprecedented in both temporal and spatial terms, requiring a response concerning the system as a whole. Accordingly, the study compares regional hospital efficiency in terms of the capacity to balance the needs of Covid-19 and non-Covid-19 patients, and then assesses the influence of regional measures implemented by actors other than hospitals to help to strike such a balance.

The findings of this study have two main practical implications for developing effective strategies for managing health-care demand during the pandemic, with implications for the post-Covid-19 phase. Health-care administrators worldwide are running into difficulty in efficiently using resources to deal with the pandemic. Reallocating resources to Covid-19 patients without the risk of a significant negative impact on non-Covid-19 patients is a fundamental objective that is often disregarded. The results of this study show that only a small number of actors manage to balance resources efficiently, but also that many actors manage to improve the balance in the medium term, reflecting a naturally incremental learning process. Second, the management of demand to control the flow of patients, matching it efficiently with available capacity, does not require a redundancy of tools but rather a targeted approach. Our findings suggest that decentralized services provided by staff working for non-critical patients outside the hospital work well. Autonomous decision-making, combined with agile measures, ensures a prompt response for less severe patients to avoid aggravating the demand for hospital beds. On the other hand, integration models that are not fully tested risk slowing down the response, giving rise to considerable costs without producing effective results.

## Data Availability

The datasets generated and/or analysed during the current study are not publicly available due to the huge amount of the text material but are available from the corresponding author on reasonable request.
